# Supplementary material for: Causal Relationship between Aging and Anorexia Nervosa: A White-Matter-Microstructure-Mediated Mendelian Randomization Analysis
Source: Biomedicines. 2024 Aug 16;12(8):1874. doi: 10.3390/biomedicines12081874 (PMC11351342; doi:10.3390/biomedicines12081874)
Supplement: Supplementary file 1 [file biomedicines-12-01874-s001.zip › Supplementary Figure/Figure S4/Singe SNP plot of MR analysis for TL to ubm-b-1844.pdf]

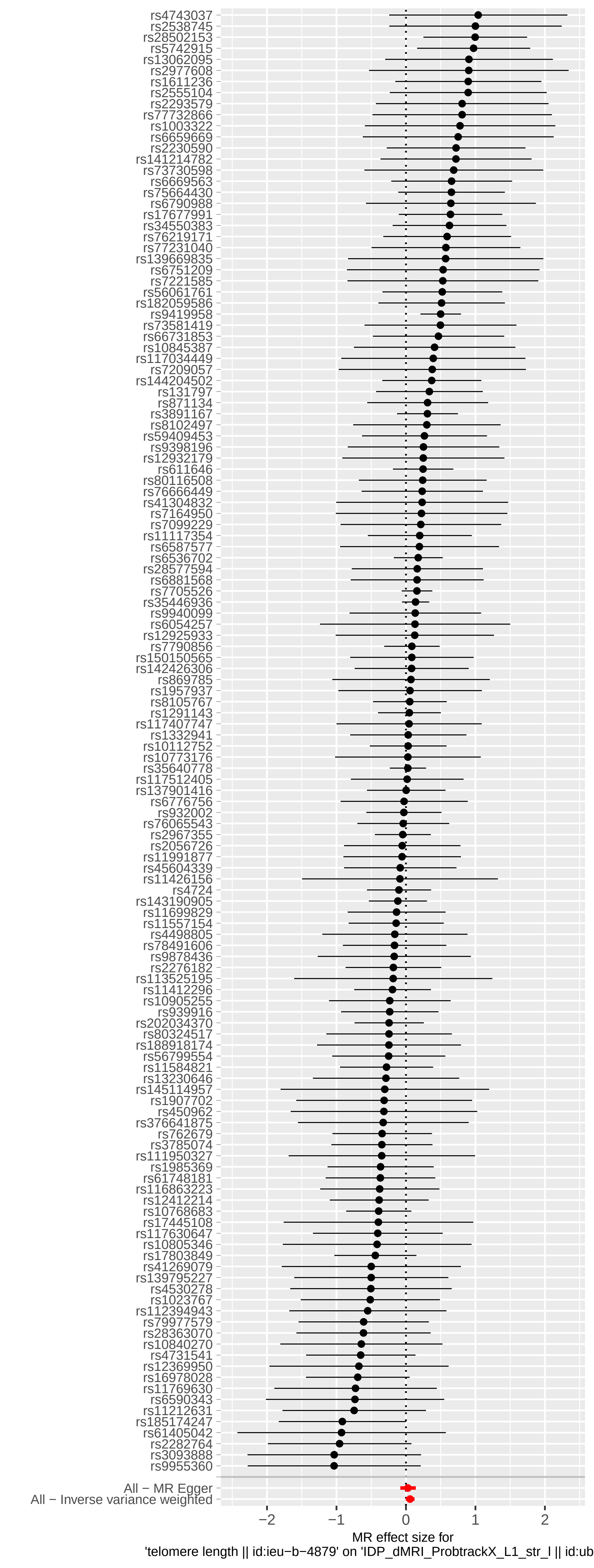
rs4743037
rs2538745
rs28502153
rs5742915
rs13062095
rs2977608
rs1611236
rs2555104
rs2293579
rs77732866
rs1003322
rs6659669
rs2230590
rs141214782
rs73730598
rs6669563
rs75664430
rs6790988
rs17677991
rs34550383
rs76219171
rs77231040
rs139669835
rs6751209
rs7221585
rs56061761
rs182059586
rs9419958
rs73581419
rs66731853
rs10845387
rs117034449
rs7209057
rs144204502
rs131797
rs871134
rs3891167
rs8102497
rs59409453
rs9398196
rs12932179
rs611646
rs80116508
rs76666449
rs41304832
rs7164950
rs7099229
rs11117354
rs6587577
rs6536702
rs28577594
rs6881568
rs7705526
rs35446936
rs9940099
rs6054257
rs12925933
rs7790856
rs150150565
rs142426306
rs869785
rs1957937
rs8105767
rs1291143
rs117407747
rs1332941
rs10112752
rs10773176
rs35640778
rs117512405
rs137901416
rs6776756
rs932002
rs76065543
rs2967355
rs2056726
rs11991877
rs45604339
rs11426156
rs4724
rs143190905
rs11699829
rs11557154
rs4498805
rs78491606
rs9878436
rs2276182
rs113525195
rs11412296
rs10905255
rs939916
rs202034370
rs80324517
rs188918174
rs56799554
rs11584821
rs13230646
rs145114957
rs1907702
rs450962
rs376641875
rs762679
rs3785074
rs111950327
rs1985369
rs61748181
rs116863223
rs12412214
rs10768683
rs17445108
rs117630647
rs10805346
rs17803849
rs41269079
rs139795227
rs4530278
rs1023767
rs112394943
rs79977579
rs28363070
rs10840270
rs4731541
rs12369950
rs16978028
rs11769630
rs6590343
rs11212631
rs185174247
rs61405042
rs2282764
rs3093888
rs9955360
All − MR Egger
All − Inverse variance weighted
−2
−1
0
1
2
MR effect size for
'telomere length || id:ieu−b−4879' on 'IDP_dMRI_ProbtrackX_L1_str_l || id:ub
